# ITPK1 Regulates Jasmonate-Controlled Root Development in *Arabidopsis thaliana*

**DOI:** 10.3390/biom13091368

**Published:** 2023-09-09

**Authors:** Naga Jyothi Pullagurla, Supritam Shome, Ranjana Yadav, Debabrata Laha

**Affiliations:** Department of Biochemistry, Division of Biological Sciences, Indian Institute of Science (IISc), Bengaluru 560012, India; nagajyothi@iisc.ac.in (N.J.P.); supritams@iisc.ac.in (S.S.); ranjanayadav@iisc.ac.in (R.Y.)

**Keywords:** inositol phosphate, ITPK1, jasmonic acid, root development, *Arabidopsis thaliana*

## Abstract

Jasmonic acid (JA) is a plant hormone that regulates a plethora of physiological processes including immunity and development and is perceived by the F-Box protein, Coronatine-insensitive protein 1 (COI1). The discovery of inositol phosphates (InsPs) in the COI1 receptor complex highlights their role in JAperception. InsPs are phosphate-rich signaling molecules that control many aspects of plant physiology. Inositol pyrophosphates (PP-InsPs) are diphosphate containing InsP species, of which InsP_7_ and InsP_8_ are the best characterized ones. Different InsP and PP-InsP species are linked with JA-related plant immunity. However, role of PP-InsP species in regulating JA-dependent developmental processes are poorly understood. Recent identification of ITPK1 kinase, responsible for the production of 5-InsP_7_ from InsP_6_ *in planta*, provides a platform to investigate the possible involvement of ITPK-derived InsP species in JA-related plant development. Here, in this study, we report that ITPK1-defective plants exhibit increased root growth inhibition to bioactive JA treatment. The *itpk1* plants also show increased lateral root density when treated with JA. Notably, JA treatment does not increase ITPK1 protein levels. Gene expression analyses revealed that JA-biosynthetic genes are not differentially expressed in ITPK1-deficient plants. We further demonstrate that genes encoding different JAZ repressor proteins are severely down-regulated in ITPK1-defective plants. Taken together, our study highlights the role of ITPK1 in regulating JA-dependent root architecture development through controlling the expression of different JAZ repressor proteins.

## 1. Introduction

Inositol phosphates (InsPs) are phosphates containing cellular messengers that control a large array of physiological processes in eukaryotes [1,2,3,4]. Combinatorial action of different kinases and phosphatases results into diverse inositol phosphate messengers of which InsP_6_ is one of the most abundant InsP species. InsP_6_, also known as phytic acid, contributes to different cellular processes either directly or indirectly by serving as a precursor for a class of signaling molecules known as inositol pyrophosphates (PP-InsP). PP-InsPs comprise of one or more diphosphate groups and are critical second messengers in yeast, amoeba and metazoan [5,6,7]. In yeast and mammals, PP-InsPs exert regulatory control over a diverse array of cellular processes. These include DNA repair, immune response, mRNA export, ribosome biogenesis, chromatin modification, encompassing telomere length, phosphate, and cellular energy homeostasis [4,6,8,9,10,11,12,13].

**Figure 1:**
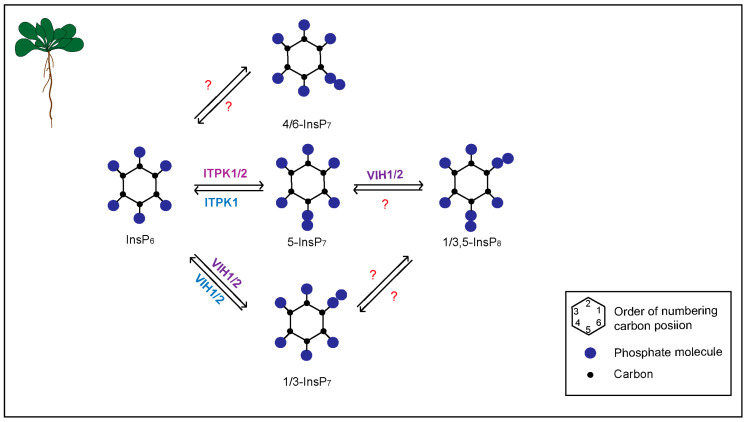
A cartoon depicting inositol pyrophosphate (PP-InsP) metabolism in *Arabidopsis*. ITPK1/2 phosphorylates InsP_6_ at position C5 to generate 5-InsP_7_. Under varying ADP/ATP ratios, ITPK1 acts as an ATP synthase and could also catalyze the ADP phosphotransferase reaction to form InsP_6_ and ATP from 5-InsP_7_. Further phosphorylation of InsP_6_ and 5-InsP_7_ by VIH1/2 results in InsP_7_ and InsP_8_, respectively; the isomeric identity of these PP-InsP species is not fully understood yet. Based on current knowledge, it is likely that VIH contributes to the synthesis of 1/3-InsP_7_ and 1/3,5-InsP_8_. The reverse enzymatic conversion of 1/3,5-InsP_8_ is catalyzed by the phosphatase domain of VIH1/2. Protein(s) responsible for the synthesis of 4/6-InsP_7_ in plants are not known currently and hence denoted by question mark.

The PP-InsP biosynthetic pathway is well established in yeast and mammals, where Kcs1/IP6K-type proteins synthesize 5-InsP_7_, and Vip1/PPIP5K-type kinases phosphorylate InsP_6_ and 5-InsP_7_, generating 1-InsP_7_ and 1,5-InsP_8_, respectively [14,15,16,17,18]. While Kcs1/IP6K-type proteins are absent in plants, Vip1/PPIP5K-type kinases are encoded by all plant genomes [19,20,21]. Similar to yeast and mammals, the *Arabidopsis* PPIP5K isoforms, VIH1 and VIH2, catalyze the synthesis of InsP_8_ and are likely involved in synthesizing 1/3-InsP_7_ *in planta* (Figure 1) [20,22,23]. Recently, ITPK1 and ITPK2 were identified to catalyze the synthesis of 5-InsP_7_ from InsP_6_ in vitro [24,25,26] and *in planta* [23,27,28]. Identification of the proteins controlling PP-InsP synthesis *in planta* created new avenues to understand the physiological processes regulated by these PP-InsP isomers.

Since the detection PP-InsP species in plants [19,20,29,30,31], different studies have elucidated the involvement of these energy-rich species as signaling molecules in regulating various physiological processes in plants [1,20,21,22,23,28,32,33,34,35,36,37,38,39]. Furthermore, recent studies have revealed that the regulation of phosphate homeostasis in plants involves various InsP and PP-InsP molecules [22,23,34,36,37,40,41,42]. This regulation is likely attributed to the InsP_8_, which facilitates the physical interaction between PHOSPHATE STARVATION RESPONSE (PHR) transcription factors and stand-alone SYG1/Pho81/XPR1 (SPX) proteins [22,23,34,42]. Intriguingly, certain bacterial plant pathogens disrupt plant hormone signaling dependent on InsP_6_ and potentially PP-InsPs, through the injection of a XopH-type effector protein, that functions as a 1-phytase [43]. However, the precise benefits for the pathogen resulting from this modulation of the host’s InsP and PP-InsP status remain unclear [43]. Moreover, recent investigations have established a connection between pathogen defense and Pi starvation, highlighting the role of InsPs and PP-InsPs as mediators of crosstalk between abiotic and biotic stresses [33].

Identification of InsP_6_ molecule in the auxin receptor, Transport Inhibition Response 1 (TIR1) complex unveils an unexplored role of inositol phosphates in the phytohormone-mediated signaling in plants [44]. Subsequent studies have established the importance of InsP homeostasis in modulating auxin responses in plants [28,45]. Inositol phosphates are also linked with jasmonic acid (JA)-dependent responses [20,46,47]. JA is a phytohormone involved in plant development and immunity [48]. Jasmonoyl-isoleucine (JA-Ile), the bioactive JA-derivative [49,50], is perceived by the Coronatine Insensitive 1 (COI1) receptor protein that plays a crucial role in regulating JA responses [51,52,53,54]. Binding of JA-Ile to COI1 facilitates its interaction with different Jasmonate ZIM Domain (JAZ) transcriptional repressor proteins and promotes degradation, which derepresses MYC2 and other transcription factors, facilitating JA-dependent gene expression [55,56,57,58,59,60]. Since TIR1 and COI1, share the 33% sequence homology [52], it raised the possibility of inositol phosphate’s presence in the COI1 complex. The crystal structure of the ASK1-COI1 receptor complex revealed distinct electron densities in the core of the solenoid structure [61]. These electron densities were likely due to individual phosphates that replaced an inositol phosphate ligand derived from the insect cells, possibly due to the high concentrations of ammonium phosphate used during crystallization [61]. Indeed, the nano-electrospray mass spectroscopy of insect-purified ASK1-COI1 protein confirmed the presence of an InsP_5_ species in the receptor complex [61]. Previous studies have implicated the role of inositol phosphates in plant wound response and disease resistance [46,47,62]. Heterologous expression of human inositol 5-phosphatase in *Arabidopsis* has resulted in altered defense gene expression and increased weight of herbivorous caterpillars [63,64]. The InsP_6_-defective mutant plants, *ipk1-1* and *ips2*, exhibit susceptibility to various pathogens including the necrotrophic fungi, *Botrytis cinerea* [46]. Moreover, investigating the *Arabidopsis ipk1-1* mutant revealed its increased sensitivity to JA [47]. Additionally, the *ipk1-1* mutant exhibited enhanced defense capabilities against herbivorous insects compared to wild-type plants [47]. Collectively, these studies provide substantial evidence supporting the involvement of inositol phosphates in the regulation of JA-dependent responses in plants. Notably, *ipk1-1* plants also exhibit increased level of InsP_5_ [2-OH] and severely reduced levels of InsP_7_ and InsP_8_ species [20,23,36,37].

To comprehensively assess the potential role of PP-InsPs on JA-dependent responses, the consequences of VIH2 loss of function with impaired InsP_8_ synthesis in plants were monitored [20]. Although *vih2* mutant plants exhibited unaltered levels of InsP_5_ [2-OH], they were susceptible to different insect herbivores and fungal necrotrophs [20,32]. Furthermore, *vih2* mutants exhibited reduced JA-dependent gene expression despite elevated JA levels [20]. Thus, the compromised resilience of *Arabidopsis vih2* mutants against herbivorous insects could be attributed to impaired JA perception rather than compromised JA production [20]. Furthermore, in vitro binding experiments using various radio-labelled InsPs revealed that higher inositol polyphosphates, namely InsP_6_ and InsP_7_, exhibit greater efficacy in binding to the ASK1-COI1-JAZ1-JA-receptor complex compared to lower InsPs [20,32]. In conclusion, it has been proposed that coincidence detection of both VIH2-dependent InsP_8_ and jasmonate, form ASK1-COI1-JAZ receptor complexes that activate the JA-dependent gene expression [20]. Future work waits to explore the possible functions of other InsP and PP-InsP species in JA-dependent physiological processes.

Analyses of the *itpk1-2* mutant plants with altered levels of various InsP and PP-InsP species [23,28,36] revealed a critical role of ITPK1 in different auxin-related processes including leaf venation, thermomorphogenic responses, and primary root elongation [28]. Notably, JA also controls root growth and development [65]. Since ITPK1 is responsible for the production of 5-InsP_7_, a precursor of InsP_8_ [23,24,28], and InsP_8_ is linked with JA-perception [20], further investigation is necessary to elucidate the potential involvement of ITPK1-derived inositol phosphate species in JA-mediated physiological processes. Here, in this study, we aimed to elucidate the role of ITPK1 in jasmonate-controlled root development. Through phenotypic analysis and molecular techniques, we monitored the alterations in root architecture upon methyl jasmonate (MeJA) application in the ITPK1-defective mutant lines. Compared to wild-type plants, the *itpk1-2* mutant exhibited increased sensitivity to jasmonate, indicating the involvement of ITPK1 in jasmonate signaling. We further investigated the impact of MeJA on ITPK1 protein levels and the expression of various jasmonate-biosynthetic genes. MeJA stimulated the expression of jasmonate-responsive genes encoding JAZ repressor proteins in wild-type seedlings; however, the expression of various *JAZs* was compromised in the ITPK1-defective lines. Collectively, our findings highlight a regulatory role of ITPK1 in jasmonate-mediated root development.

## 2. Materials and Methods

### 2.1. Arabidopsis Plant Material and Growth Conditions

Seeds of T-DNA insertion lines of *Arabidopsis thaliana* (ecotype Col-0) were obtained from the Arabidopsis Biological Resource Center at Ohio State University (http://abrc.osu.edu (accessed on 1 September 2023)). The *itpk1-2* plants and the *itpk1-2* transgenic lines expressing the genomic ITPK1 fragment used in this study were reported previously [28]. Wild-type and all relevant transgenic lines were amplified together on soil and perlite mix under identical conditions (16 h light (22 °C) and 8 h dark (20 °C), and 120 µmol^−1^ m^−2^ light intensity). For sterile growth, seeds were surface sterilized in solution containing 70% (*v*/*v*) ethanol and 0.05% (*v*/*v*) Triton X-100 for 10 min and washed twice with 90% (*v*/*v*) ethanol. Sterilized seeds were sown on solidified half-strength Murashige and Skoog (MS) media (0.8% phytagel) supplemented with 1% (*w*/*v*) sucrose and then stratified for 2 days at 4 °C, and grown in a Percival plant chamber under conditions of 8 h light (22 °C) and 16 h dark (20 °C).

### 2.2. Primary Root Length and Lateral Root Density Assay

For primary root length and lateral root density analysis, seeds were sown on solidified half-strength MS media, supplemented with 1% (*w*/*v*) sucrose. After 3 days of stratification, the seedlings were grown vertically for 4 days in a Percival plant chamber under conditions of 8 h light (22 °C) and 16 h dark (20 °C). Then, seedlings were moved onto modified half-strength MS media containing 1% (*w*/*v*) sucrose. This media was either supplemented with 50 µM MeJA (dissolved in 95% ethanol which was diluted to 50 mM with distilled water) or an equal volume of ethanol was used as the mock control (0 µM MeJA). The seedlings were allowed to grow vertically for a further 7 days, and the primary root length was noted at intervals of 3, 5 and 7 days of growth. Images were taken using Bio-Rad ChemiDoc Version 2.4.0.03. The primary root length and lateral roots were quantified using ImageJ 1.53k software.

### 2.3. RT-PCR Analyses

9-day-old seedlings were harvested in liquid nitrogen for total RNA extraction using TRIzol reagent (Sigma Aldrich, St. Louis, MO, USA). The seedlings were crushed with pestle and 1 mL of TRIzol reagent was added to homogenize the seedlings with brief vortex. The homogenate was incubated at room temperature for 5 min. Chloroform (200 μL/mL) was then added to the homogenate, shaken vigorously by hand for 15 s followed by incubation of 2 min at room temperature. The homogenate was spun down at 12,000× *g* for 15 min at 4 °C for phase separation. The separated aqueous phase of the samples was taken out in a new tube. To precipitate RNA, 250 μL of 100% isopropanol was added to the aqueous phase, incubated at room temperature for 10 min, and then centrifuged at 10,000× *g* for 10 min at 4 °C. The supernatant was carefully removed from the tube, without disturbing the RNA pellet. The RNA pellet was washed with 1 mL of 75% (*v*/*v*) ethanol and vortexed briefly. The tube was centrifuged at 7000× *g* for 5 min at 4 °C, the supernatant was discarded, and the RNA pellet was air dried for 5–10 min at room temperature. Extracted total RNA was then subjected to DNase1 treatment to remove genomic DNA contamination. A total of 5–10 µg of RNA was treated with DNase1 enzyme (1 U/µL; Thermo-scientific, Waltham, MA, USA). The reactions were incubated at 37 °C for 2 h. DNase1 inactivation was performed by adding 10 µL of 50 mM EDTA at 65 °C for 10 min. RNA was precipitated by adding 250 µL of 100% ethanol and 10 µL of 3 M sodium acetate. The RNA pellet was washed with 1 mL of 75% (*v*/*v*) ethanol and centrifuged at 7000× *g* for 5 min at 4 °C. The supernatant was discarded, and RNA pellets were air dried for 5–10 min. A total of 1 µg of RNA was used to synthesize cDNA with PhiScript™ cDNA Synthesis Kit (dx/dt). The cDNA samples were diluted to 150 ng/μL of which 0.5 µL was used as template for reaction. The qPCR was performed using the DyNAmo ColorFlash SYBR Green qPCR Kit (Thermo-scientific) with CFX96 Touch Real-Time PCR Detection System (Bio-Rad Hercules, CA, USA) according to the manufacturer’s protocol (Bio-Rad). The relative quantitation method (ΔΔCT) was used to evaluate quantitative variation among replicates. *β-TUBULIN* was used as reference gene. The primers used for qPCR analyses are detailed in Supplemental Table S2.

### 2.4. Western Analyses

For immunoblot analyses, 9-day-old seedlings of *itpk1-2* lines, expressing genomic ITPK1 fragment with C-terminal G3GFP fusion (complementary line #7 and complementary line #15), were grown on sterile solidified (0.8% (*w*/*v*) phytagel), half-strength MS media, supplemented with 1% (*w*/*v*) sucrose for 7 days. The seedlings were then transferred to liquid half-strength MS media (pH 5.7), supplemented with 1% (*w*/*v*) sucrose and with or without 50 µM MeJA, and subsequently incubated for indicated time points before harvesting. Seedlings were ground to fine powder in a pre-chilled mortar in presence of liquid nitrogen and homogenized (*w*/*v*) in protein extraction buffer [5 mM Tris–HCl, pH 7.5, 150 mM NaCl, 10 mM MgCl_2_, 1 mM EDTA] containing 1× plant protease inhibitor (Sigma). The extracts were incubated for 10 min at 4 °C and centrifuged at high spin for 10 min at 4 °C. The concentration of protein was measured using Bradford assay. Equal concentration of protein was used for the Western blot analysis. Protein extracts were boiled with loading buffer containing 6XSDS dye for 10 min, and were resolved by SDS-PAGE and electrophoretically transferred onto nitrocellulose membrane. Following electroblotting, the membrane was blocked for 30 min in 5% (*w*/*v*) skimmed milk prepared in PBS buffer (6.5 mM Na_2_HPO_4_, 1.5 mM KH_2_PO_4_, 3 mM KCl, 0.15 M NaCl, pH 7.4) containing 0.5% (*v*/*v*) Triton X-100 and then incubated overnight at 4 °C with a mouse anti-GFP (dilution 1:1000) as primary antibody (Roche). The immunoreactive bands were detected with HRP-conjugated goat-anti-mouse IgG (dilution 1:5000) as secondary antibody (ImmunoTag) using the peroxidase substrate, 4-chloro-1-naphthole (Bio-Rad). The same membrane was re-detected with anti-ACTIN (Sigma, A0480) to normalize the protein loading, as indicated. Densitometric analysis of the ITPK1 protein band has been performed using Image J software.

## 3. Results

### 3.1. Arabidopsis itpk1-2 Lines Exhibit Enhanced Jasmonate-Mediated Root Growth Inhibition and Increased Lateral Root Formation

**Figure 2:**
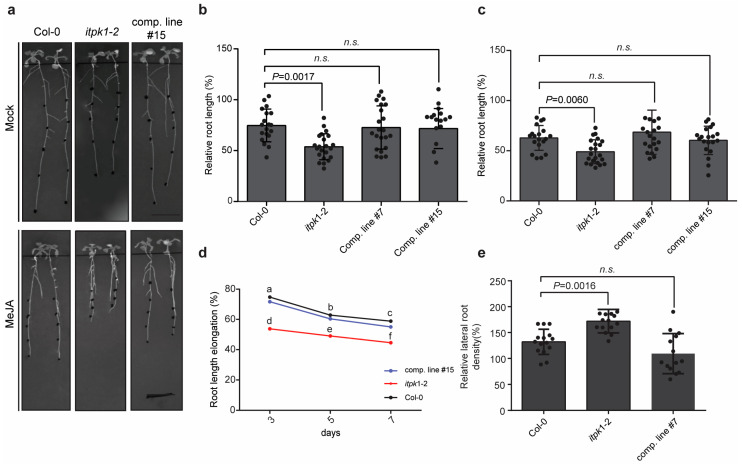
Sensitivity of *itpk1-2* plants to methyl jasmonate (MeJA) treatment. (**a**) Representative pictures of seedlings of wild-type (Col-0), *itpk1-2* and one complemented *itpk1-2* line grown in presence or absence of MeJA. Seeds were germinated on solidified half-strength MS agar media, supplemented with 1% (*w*/*v*) sucrose. After 7 days, seedlings were transferred to solidified half-strength MS media supplemented with 1% (*w*/*v*) sucrose and 50 µM MeJA. The percentage of changes in primary root length induced by 50 µM MeJA was determined for the wild-type, *itpk1-2* mutant, and the selected complemented lines. The growth of root length was evaluated after (**b**) 3 days and (**c**) 5 days using ImageJ software. The data presented are means ± SD (*n* ≥ 20). The *p* values depict the significance in two-way ANOVA followed by Tukey’s test. (**d**) The relative root elongation of the designated genotypes under 50 µM MeJA treatment. Different letters indicate significance in two-way ANOVA followed by Tukey’s test (a and d, *p* < 0.01; b to e, *p* < 0.05; c to f, *p* < 0.05). The experiment was repeated multiple times with consistent results (Supplemental Table S1). (**e**) The lateral root density of the designated genotypes grown in 50 µM MeJA was quantified after 7 days. Lateral roots were quantified using ImageJ software. Images were taken using Bio-Rad ChemiDoc. The data presented are means ± SD (*n* ≥ 20). The *p* values depict the significance and *n.s.* depicts the non-significance in two-way ANOVA followed by Tukey’s test.

**Figure 3:**
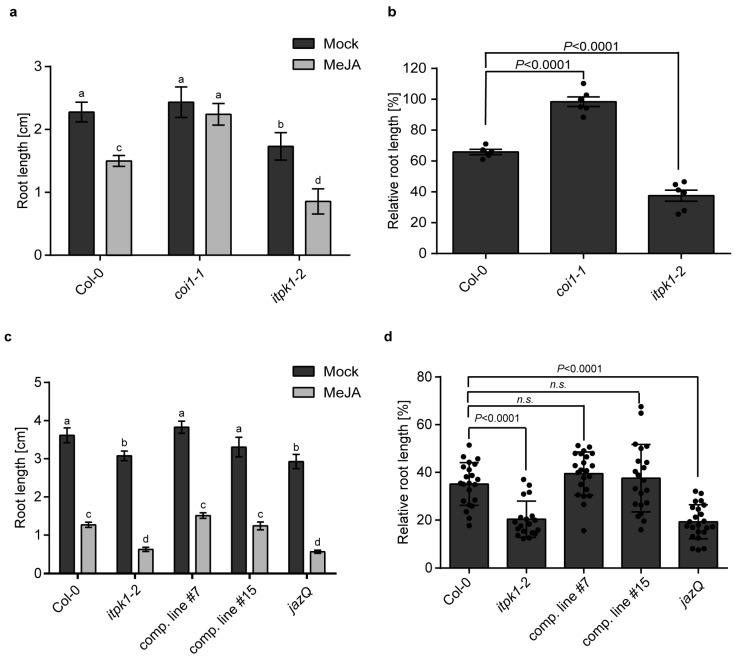
ITPK1-defective plants exhibit altered sensitivity to exogenous MeJA application. Seeds were germinated on solidified half-strength MS agar media, supplemented with 1% (*w*/*v*) sucrose. The homozygous *coi1-1* plants were identified from heterozygous seed population by selecting the MeJA-insensitive seedlings. After 7 days, seedlings were transferred to solidified half-strength MS media supplemented with 1% (*w*/*v*) sucrose and 50 µM MeJA. (**a**,**c**) The changes in primary root length induced by 50 µM MeJA was determined for the designated genotypes after 5 days using ImageJ software. The data presented are means ± SD (*n* ≥ 5). (**a**) Different letters indicate significance in in two-way ANOVA followed by Dunnett’s test (a and b, *p* < 0.001; a to c, *p* < 0.0001; b to d, *p* < 0.0001). (**c**) Different letters indicate significance in in two-way ANOVA followed by Dunnet t’stest (a and b, *p* < 0.03; a to c, *p* < 0.009; a to d, *p* < 0.002). (**b**,**d**) The relative root elongation of the designated genotypes after 5 days of 50 µM MeJA treatment. The data presented are means ± SD (*n* ≥ 20). The *p* values depict the significance and *n.s.* depicts the non-significance in two-way ANOVA followed by Tukey’s test. Images were taken using Bio-Rad ChemiDoc system.

To investigate whether JA-mediated processes are altered in the ITPK1-deficient plants, we monitored root development [65,66] and lateral root (LR) formation [67] after exogenous application of methyl jasmonate (MeJA), a bioactive form of jasmonate [49]. The impact of MeJA treatment on root development was assessed for wild-type, *itpk1-2* and *itpk1-2::AtITPK1G3GFP* complementary lines (Figure 2, Figure 3 and Figure S1 and Table S1). Notably, primary root growth inhibition was more pronounced in *itpk1-2* plants when compared with the wild-type seedlings (Figure 2a–d, Figure 3 and Figure S1 and Table S1). Furthermore, LR density was significantly increased in the *itpk1-2* plants after MeJA treatment (Figure 2e). The altered root architecture of ITPK1-defective plants was rescued in the *itpk1-2* lines expressing the ITPK1-G3GFP translational fusion under the control of its endogenous promoter. To get further insight, we analyzed the coronatine-insensitive *coi1-1* mutant, known for its insensitivity to JA [52] and the *jaz quintuple* mutant, *jazQ*, which carries T-DNA insertions in five *JAZ* genes (*JAZ1/3/4/9/10*) and is hypersensitive to JA treatment [68]. In line with the previous observation, our analyses reveal that the *coi1-1* mutant displayed insensitivity, while the *jazQ* mutant exhibited hypersensitivity to 50 µM MeJA application (Figure 3). As shown in Figure 3a,d, the *itpk1-2* line exhibits root development similar to that of *jazQ* mutant plants. Collectively, these data suggest that *itpk1-2* plants are more sensitive to JA treatment and are defective in JA-dependent root architecture development.

### 3.2. Effect of Methyl Jasmonate (MeJA) on ITPK1 Protein Level and in the Expression of Several Jasmonate-Biosynthetic Genes

**Figure 4:**
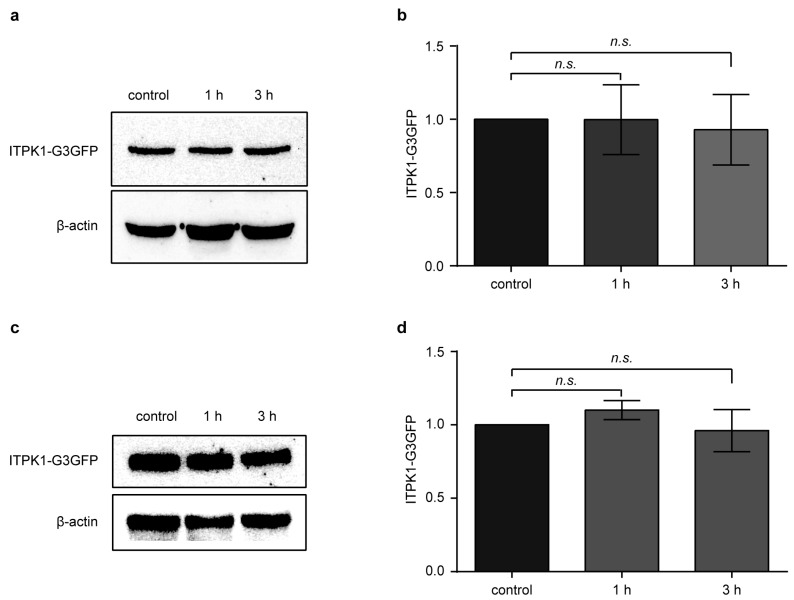
Effect of methyl jasmonate (MeJA) on the expression of ITPK1 protein. Western blot analysis of total protein extract prepared from 9-day-old (**a**) complementary line # 7 and (**c**) complementary line # 15 seedlings expressing ITPK1 in translational fusion with N-terminal G3GFP. The 9-day-old seedlings were transferred to liquid half-strength MS media (pH 5.7), supplemented with 1% (*w*/*v*) sucrose and with or without 50 µM MeJA, and then incubated for indicated time points before harvesting. Approximately 25–30 µg of protein was loaded in each well, and ITPK1 was detected with antibodies against GFP (Roche). The β-actin (45 kDa) was used as an internal loading control. Quantification of the relative ITPK1 protein levels of 9-day-old (**b**) complementary line # 7 and (**d**) complementary line # 15 seedlings following treatment of MeJA for 1 h and 3 h. Relative ITPK1 protein levels were quantified using ImageJ software. The data presented are means ± SD (*n* = 3). The *n.s.* depicts the non-significance in one-way ANOVA followed by Tukey’s test. The experiments were repeated three times with similar results.

Since *itpk1-2* plants show altered JA response, we wanted to investigate whether MeJA treatment had an influence on the ITPK1 protein level. Our findings revealed that there was no observable difference in the content of the ITPK1 protein after MeJA treatment (Figure 4, File S1). The lack of difference in the ITPK1 protein level upon treatment indicates that MeJA does not exert regulatory control over the expression of ITPK1 in plants.

**Figure 5:**
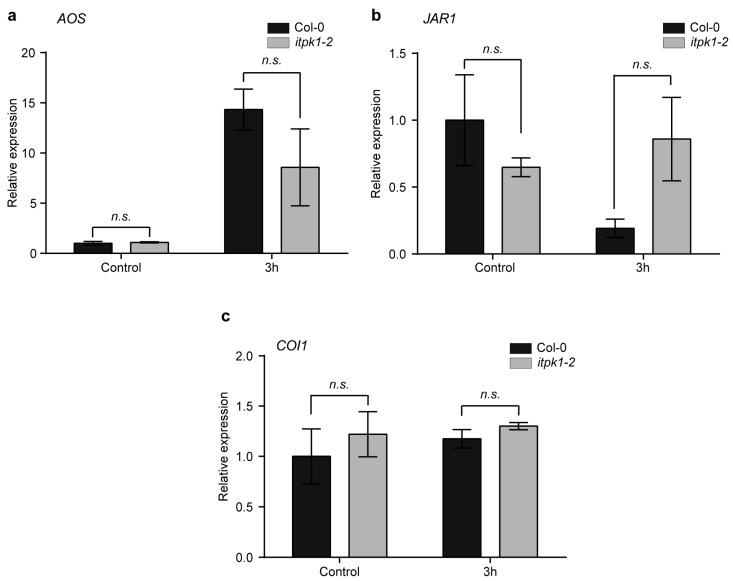
The relative expression levels of jasmonate-related genes. (**a**) *AOS* (**b**) *JAR1* and (**c**) *COI1* were assessed in seedlings of the wild-type and *itpk1-2* mutant. Seven-day-old seedlings were harvested at specified time points following methyl jasmonate (MeJA) application, along with the untreated plants. The reference gene used for normalization was *β-TUBULIN*. The results are presented as means ± SD (*n* = 3). The *n.s.* depicts the non-significance in two-way ANOVA followed by Tukey’s test. The experiments were repeated with similar results.

Furthermore, to get mechanistic insight into the role of ITPK1 in JA-mediated root development, we performed qPCR analyses to study the expression levels of various genes related to jasmonate biosynthesis and perception in wild-type and *itpk1-2* plants. For JA biosynthetic markers, we used *AOS* and *JAR1*. The ALLENE OXIDE SYNTHASE (*AOS*) gene encodes a key enzyme that generates the first committed precursor, 12-oxo-phytodienoic acid (cis-OPDA), that is converted into JA by OPDA REDUCTASE 3 (OPR3) and β-oxidation in peroxisomes [50,69]. JASMONATE RESISTANT 1 (*JAR1*) gene encodes a member of the GH3 family enzyme responsible for forming JA-Ile from JA [70], and the COI1 is the core member of the jasmonate signaling pathway, which encodes a receptor of JA [51,52,53,54]. Furthermore, we also monitored *MYC2* expression, a member of the bHLHzip family of transcription factors, which is a component of the JA signaling pathway that regulates JA-mediated wounding- and pathogen-defense-related genes [71]. After treatment with 50 µM MeJA, no significant differences were observed in the expression level of any of these genes between the wild-type and *itpk1-2* plants (Figure 5 and Figure S2). Collectively, these results indicate that ITPK1 does not play a role in modulating the expression of these genes during JA-dependent root development.

### 3.3. ITPK1 Is Required for the Regulation of Methyl Jasmonate (MeJA) Induced JAZ Transcript Levels

**Figure 6:**
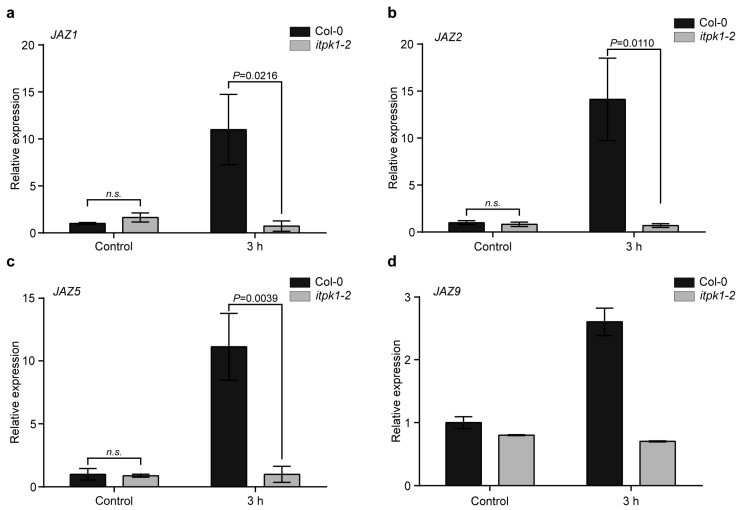
ITPK1 controls MeJA-dependent expression of (**a**) *JAZ1* (**b**) *JAZ2* (**c**) *JAZ5*, and (**d**) *JAZ9* genes. Seven-day-old seedlings were harvested at specified time points following methyl jasmonate (MeJA) application, along with the untreated plants. The relative transcript abundance was calculated by normalizing to the reference gene, *β-TUBULIN*. The data are presented as means ± SD (*n* = 3). The *P* values depict the significance and *n.s.* depicts the non-significance in two-way ANOVA followed by Tukey’s test. The qPCR analyses were repeated independently with similar results.

JAZ proteins are critical repressors of the jasmonate signaling pathway [56,57,61]. The *jazQ* mutant seedlings lacking five JAZ repressors show an increased root growth inhibition to exogenous MeJA treatment when compared with the wild-type seedlings (Figure 3) [68]. Hence, we investigated whether the absence of ITPK1 would affect the expression of genes encoding different JAZ repressors. As anticipated, the wild-type plants exhibited increased *JAZ1* expression after MeJA treatment, whereas *itpk1-2* plants showed a robust decrease in the expression of *JAZ1* (Figure 6a). To investigate whether the expression of genes encoding other JAZ proteins is affected in ITPK1-deficient plants, we monitored the expression of *JAZ2*, *JAZ5*, and *JAZ9* after MeJA treatment (Figure 6). Notably, the expression of these *JAZs* is severely compromised in the ITPK1-deficient lines. Collectively, our findings suggest that ITPK1 is critical for MeJA-induced *JAZ* expression.

## 4. Discussion

Jasmonic acid is a critical signaling compound that regulates varied physiological processes in plants, such as root growth inhibition, anthocyanin accumulation, and stress responses [48,72,73,74,75]. Inositol phosphates have been shown to regulate jasmonate signaling pathways [20,46,62], but it is still largely unclear whether a specific InsP isomer mediates certain JA responses or different InsP species work collaboratively to modulate specific JA processes in plants. Interestingly, our recent study found that 5-InsP_7_-deficient *itpk1-2* plants exhibit short root phenotypes [28] As discussed previously, JA plays a critical role in regulating root growth and development [65,66]. Given that ITPK1 is responsible for the production of 5-InsP_7_, which serves as a precursor to InsP_8_, and considering the association of InsP_8_ with JA perception [20], this underscores the need for further investigation to uncover the potential contribution of inositol phosphate species derived from ITPK1 in JA-mediated root development.

In this current study, we have demonstrated that the function of ITPK1 is critical for regulating jasmonate responses. The *itpk1-2* mutant plants show enhanced lateral root formation and increased primary root inhibition after MeJA treatment (Figure 2 and Figure 3). The pronounced effect of MeJA on *itpk1-2* root architecture suggests that *itpk1-2* mutant plants are more sensitive to MeJA treatment and hence, that ITPK1 acts as a negative regulator in the JA-dependent root development. Our experiments suggest that ITPK1 levels remain unaltered after MeJA treatment (Figure 4); however, further work is required to unravel any post-translational modification of ITPK1 after the hormone treatment. Based on the gene expression analysis, we have concluded that the expression levels of JA biosynthetic genes were not influenced or regulated by ITPK1 during JA-dependent root development (Figure 5). Taken together, our results imply that other factors or regulatory mechanisms are involved in ITPK1-mediated JA responses. Future research should determine the JA and its derivative both in wild-type and *itpk1* mutant plants.

The jasmonate signaling pathway involves extreme transcriptional reprogramming involving intricate interactions between both positive and negative regulators. The JAZ repressors are responsible for modulating the jasmonate signaling by interacting with various transcription factors [49,72,76,77,78]. Compromised expression of various *JAZ*s after MeJA treatment in the ITPK1-deficient plants (Figure 6) highlights the importance of ITPK1 activity in maintaining cellular *JAZ* levels. Overall, these findings further suggest that the hypersensitivity phenotype of *itpk1-2* mutant plants to MeJA could be due to the reduced *JAZ* expression in these mutant plants. However, comparative analyses of different JAZ protein levels between the wild-type and ITPK1-deficient plants will further corroborate the role of ITPK1 in regulating JAZ repressor levels. Together, the findings of this study provide insights into the role of ITPK1 in JA-mediated root development. Further investigations are required to explore the mechanism underlying the altered root development phenotype of *itpk1-2* upon MeJA treatment. Future studies should clarify whether compromised catalytic activity of ITPK1-defective plants is responsible for the *itpk1*-associated root development defects. It will be interesting to examine the possibility of different inositol phosphates forming a series of distinctive JA co-receptor complexes and the physiological importance of such diverse co-receptor complexes in plants. Understanding the role of ITPK1 in modulating auxin and JA signaling might clarify its involvement in mediating crosstalk between the two phytohormones.

It is worth noting that different phytohormones other than jasmonate, including ethylene, auxin, abscisic acid (ABA), gibberellin (GA), cytokinin (CK), strigolactone (SL), and brassinosteroid (BR), have been shown to exert essential control over primary root growth [79,80,81,82]. In our previous study, we reported that ITPK1-defective plants are rather insensitive to auxin treatment [28]. In this study, we found that *itpk1* plants are more sensitive to MeJA treatment when compared with wild-type plants, highlighting the role of ITPK1 in the differential regulation of phytohormones. Whether ITPK1 contributes to other phytohormone-dependent root development is yet to be addressed.

## 5. Conclusions

Overall, this study provides insights into the involvement of ITPK1 in JA-dependent root development and sheds light on the role of ITPK1-derived InsP species in hormone signaling. Furthermore, our results highlight the importance of ITPK1 in JA-induced *JAZ* expression. These findings pave the way for further exploration of the role of various InsP and PP-InsP species in regulating JA responses.

## Data Availability

The data that support the findings of this study are all provided within the body of the manuscript.

## Supplementary Materials

The following supporting information can be downloaded at https://www.mdpi.com/article/10.3390/biom13091368/s1, File S1: Original images of Western blotting; Figure S1: Sensitivity of *itpk1-2* plants to methyl jasmonate (MeJA) treatment; Figure S2: The relative expression level of *MYC2* in seedlings of the wild-type and *itpk1-2* mutant plants; Table S1: Data analyses of three independent experiments presented in Figure 2 and Figure 3; Table S2: List of primers used for qPCR analyses.
