# Supplementary material for: ITPK1 Regulates Jasmonate-Controlled Root Development in Arabidopsis thaliana
Source: Biomolecules. 2023 Sep 9;13(9):1368. doi: 10.3390/biom13091368 (PMC10526342; doi:10.3390/biom13091368)

Supplementary File S1. Uncropped blots. Original western blot images of total protein extract prepared from 9-day-old complementary line # 7 (Figure 1) and complementary line # 15 (Figure 2) seedlings expressing ITPK1 in translational fusion with N-terminal G3GFP.

Figure 1

a. Exp1. Blot probed with Anti-ACTIN for ACTIN (control) Exp1. Blot probed with Anti-GFP for ITPK1

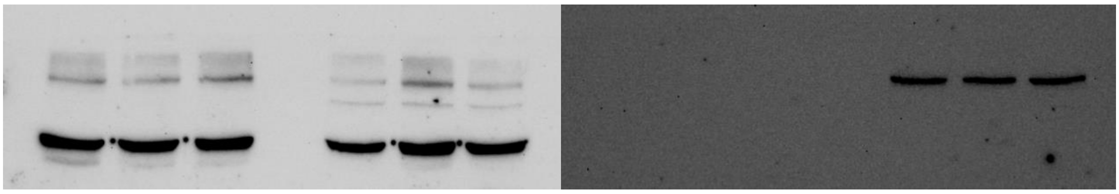

Merge images of above blots with ponceau images of Blot

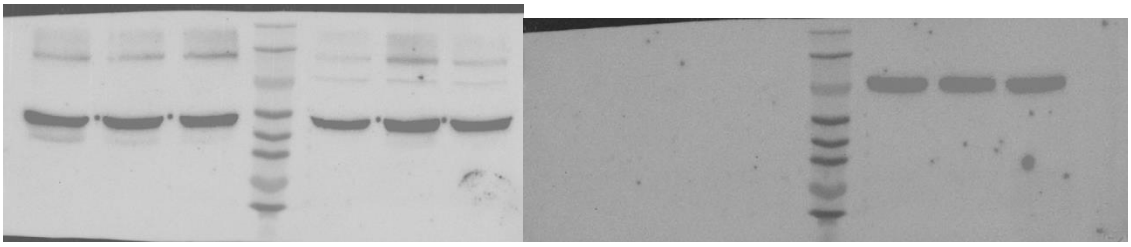

b. Exp2. Blot probed with Anti-ACTIN for ACTIN (control) Exp2. Blot probed with Anti-GFP for ITPK1

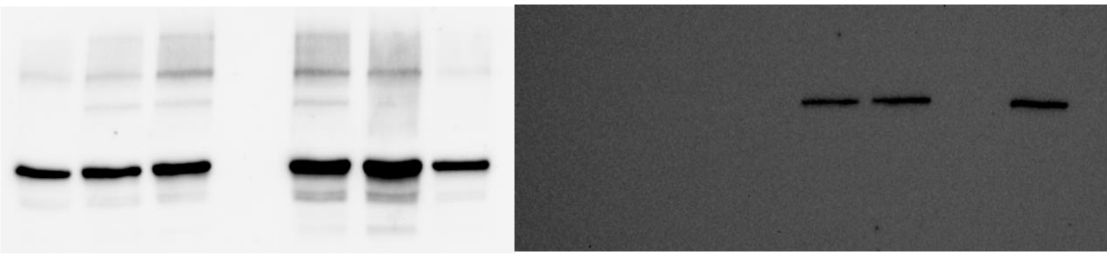

c. Exp3. Blot probed with Anti-ACTIN for ACTIN (control) Exp3. Blot probed with Anti-GFP for ITPK1

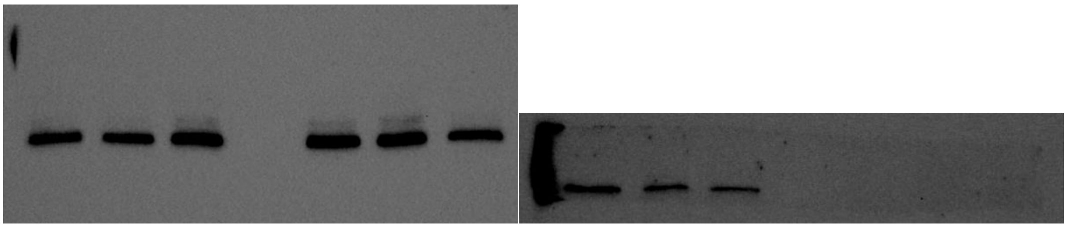

Figure 2

a. Exp1. Blot probed with Anti-ACTIN for ACTIN (control)

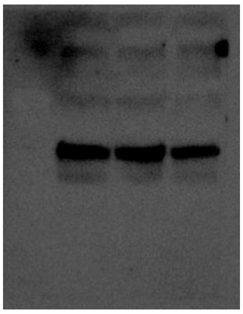

Exp1. Blot probed with Anti-GFP for ITPK1

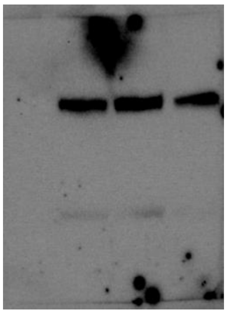

b. Exp2. Blot probed with Anti-ACTIN for ACTIN (control)

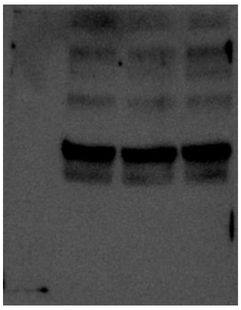

Exp2. Blot probed with Anti-GFP for ITPK1

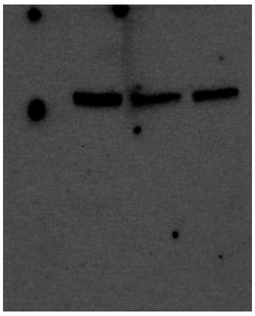

c. Exp3. Blot probed with Anti-ACTIN for ACTIN (control)

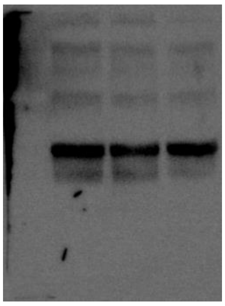

Exp3. Blot probed with Anti-GFP for ITPK1

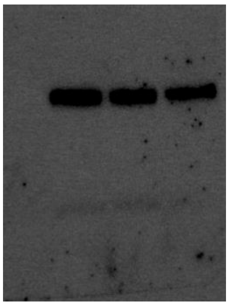

Supplement: Supplementary file 1 [file biomolecules-13-01368-s001.zip › biomolecules-2482938-supplementary/biomolecules-2482938-figures.pdf]
