# Supplementary material for: ITPK1 Regulates Jasmonate-Controlled Root Development in Arabidopsis thaliana
Source: Biomolecules. 2023 Sep 9;13(9):1368. doi: 10.3390/biom13091368 (PMC10526342; doi:10.3390/biom13091368)
Supplement: Supplementary file 1 [file biomolecules-13-01368-s001.zip › biomolecules-2482938-supplementary/biomolecules-2482938-supplementary.pdf]

Supplementary Material

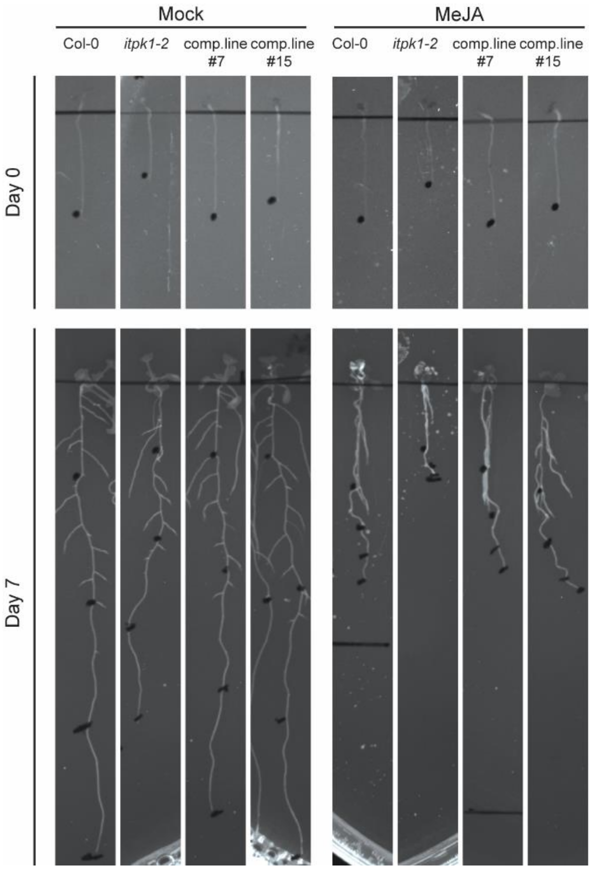

Figure S1. Sensitivity of *itpk1-2* plants to methyl jasmonate (MeJA) treatment. (**a**) Representative pictures of seedlings of wild-type (Col-0), *itpk1-2* and two complemented *itpk1-2* lines grown in presence or absence of MeJA. Seeds were germinated on solidified half-strength MS agar media, supplemented with 1% (w/v) sucrose. After 7 days, seedlings were transferred to solidified half-strength MS media supplemented with 1% (w/v) sucrose and 50 µM MeJA. The seedlings were allowed to grow vertically for further 7 days and the primary root length was noted at intervals of 3, 5 and 7 days of growth. The primary root length was quantified using ImageJ software. Images were taken using chemiDoc.

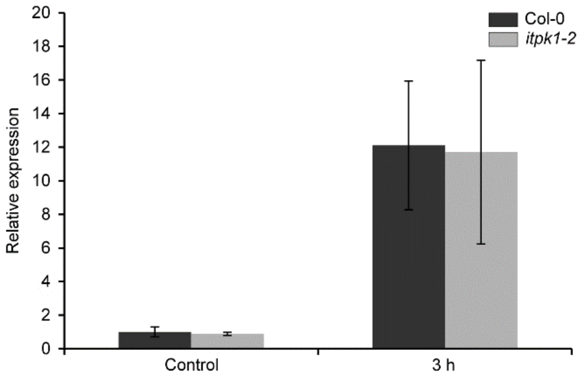

Figure S2- The relative expression level of *MYC2* in seedlings of the wild-type and *itpk1-2* mutant. 7-day-old seedlings were harvested at specified time points following MeJA application, along with the untreated plants. *β-TUBULIN* was used as reference gene. Values are means ± SE from three independent biological replicates.

Table S1. Comparison and statistical results of three independent experiments.

			Relative root length		
			Mean	STDEV	SE
Experiment 1	3DAYS	Col-0	35.53	11.21	2.29
		itpk1-2	25.41	8.44	1.80
		compl.line#7	43.59	9.23	1.88
		compl.line#15	40.32	16.01	3.08
	5DAYS	Col-0	35.13	8.74	1.86
		itpk1-2	20.39	7.39	1.70
		compl.line#7	39.50	8.87	1.89
		compl.line#15	37.58	13.82	3.01
Experiment 2	3DAYS	Col-0	74.73	15.62	3.58
		itpk1-2	53.76	12.46	2.66
		compl.line#7	72.74	20.74	4.42
		compl.line#15	71.70	19.15	4.18
	5DAYS	Col-0	62.81	12.09	2.77
		itpk1-2	49.04	11.58	2.47
		compl.line#7	68.52	21.50	4.69
		compl.line#15	60.39	13.84	3.09
Experiment 3	3DAYS	Col-0	37.2	7.9	1.81
		itpk1-2	27.67	5.55	1.43
		compl.line#7	40.54	9.98	2.35
		compl.line#15	33.17	5.36	1.3
	5DAYS	col-0	30.5	5.88	1.38
		itpk1-2	23.05	5.06	2.26

Table S2. List of primers used for qPCR analyses.

AOS qPCR_F	GTGAAATGCTTTACGGTTATC
AOS qPCR_R	ACCAAAACAACAAAATCCTTAC
JAR1-qPCR_F	AGCTGCTCACACCTAACCCTG
JAR1-qPCR_R	TACATTTGGTGCGGATCGTC
COI1-qPCR_F	GGTTGTTGCTTCAGTGAGCG
COI1-qPCR_R	GCTCGATGTTCCAGTACGGT
JAZ1 qPCR_F	TGCGATCCAGCCAAAGCGTCT
JAZ1 qPCR_R	AACCGAGCCACGACATGTTGCC
*JAZ9*_qPCR_F	AGCTCGGAAAGCATCCTTGGCT
*JAZ9*_qPCR_R	TGCACTCATAAGCCTCTCTTTGCG
MYC2 qPCR_F	TTGCTCCGTCGGATGACGCT
MYC2 qPCR_R	AATCCCGCACCGCAAGCGAA
PP2AA3 qPCR_F	GGCAGAAGTTCGGATAGCAG
PP2AA3 qPCR_R	CAATGCAGATCTGACGTGCT
*JAZ2*_qPCR_1_F	CTTCACTTCATCGGTTCCTTGAGAAGAG
*JAZ2*_qPCR_1_R	CGTGAACTGAGCCAAGCTGGGT
*JAZ5*_qPCR_1_F	GCAGGGCATTCCAAAGGCGA
JAZ5 _qPCR_1_R	AGCTGCGAACTTCCTGGCTGT
*Beta-Tubulin*_qPCR_F	GTCTGGTGTGACTTGCTGTC
*Beta-Tubulin*_qPCR_R	ACGGTACTGCTGAGAACCTC
